# A Comparative Study of Antioxidative Activity of Saliva in Children and Young Teenagers with and without Gingivitis

**DOI:** 10.3390/medicina57060569

**Published:** 2021-06-03

**Authors:** Olivera Tričković Janjić, Tatjana Cvetković, Branislava Stojković, Raša Mladenović, Mila Janjić Ranković

**Affiliations:** 1 Department of Preventive and Pediatric Dentistry, Faculty of Medicine, University of Niš, 18000 Niš, Serbia; branislava.stojkovic@medfak.ni.ac.rs; 2 Department of Biochemistry, Faculty of Medicine, University of Niš, 18000 Niš, Serbia; tanja_cvetkovic@yahoo.co.uk; 3 Department for Dentistry, Faculty of Medicine, University of Priština, 38220 Kosovska Mitrovica, Serbia; rasa.mladenovic@med.pr.ac.rs; 4 Department of Orthodontics and Dentofacial Orthopedics, University Hospital, LMU Munich, 80336 Munich, Germany

**Keywords:** children, teenagers, gingivitis, saliva, total antioxidant capacity, catalase, glutathione peroxidase

## Abstract

*Objectives*: The aim of this study was to compare the values of total antioxidant capacity (TAC), catalase (CAT) and glutathione peroxidase (GPX) in the saliva of children and young teenagers with and without gingivitis. *Materials and Methods*: A total of 120 children and young teenagers of the mean age of 12.2 participated in the research. Gingival condition was assessed using the Löe and Silness Gingival Index. The subjects were divided into groups of those without gingivitis and those with gingivitis. Samples of unstimulated saliva were collected, and TAC, CAT and GPX were determined spectrophotometrically. *Results*: By comparing the values of TAC, CAT and GPX in subjects with and without gingivitis, significantly lower values of TAC (*p* < 0.001) and CAT (*p* < 0.001) were observed in the group of subjects with gingivitis. The correlation analysis of these values showed a positive correlation in groups of subjects not suffering from gingival inflammation and those with gingival inflammation. *Conclusions*: The study showed significantly lower values of TAC and CAT in the saliva of subjects with gingivitis. This indicates their possible role as a potential biomarker in the early diagnosis and expression of periodontal disease in children and young teenagers.

## 1. Introduction

Free radicals and oxidative stress play a significant role in the etiopathogenesis of many diseases, such as inflammatory, allergic, metabolic, malignant diseases, and even in a physiological process such as aging [1,2,3,4]. As protection against the possible harmful effects of free radicals, a specific defense system, known as the antioxidant system, has been established in the body [5]. The activity of the antioxidant system depends on the type of the caused oxidative stress and the nature of the affected organ [6,7].

With its antioxidant system, saliva plays a significant role in defending oral tissues from the harmful effects of free radicals [8,9]. Oxidative stress occurs when there is an existing imbalance between the production of free radicals and the ability of salivary antioxidants to neutralize them. This can be the biochemical basis for the development of oral diseases, most often the ones affecting the periodontium [10,11]. Therefore, the values of prooxidative parameters or antioxidant conditions of the environment in which the process occurs might serve as important biomarkers for the early identification of potential tissue damage [8,12,13].

Saliva protects oral tissues from the harmful effects of free radicals with numerous antioxidants present in its composition, including catalase (CAT) and glutathione peroxidase (GPX) [14,15,16]. Catalase is an enzymatic antioxidant active in the detoxication of oral tissues from hydrogen peroxide, which significantly increases the antioxidant capacity of saliva [17,18]. Increased CAT activity in the saliva has been proven in all inflammatory processes in the oral cavity [19]. A sudden and short-term increase in the CAT activity in the saliva has also been observed in some physiological conditions with an increased metabolic activity, owing to physical activity [20]. As intense oxidative stress primarily leads to CAT elevation; CAT can be considered an early marker of disease onset [21]. Glutathione tripeptide is required for GPX activity, enabling the neutralization of hydrogen peroxide, lipid peroxide and other peroxides [22]. Glutathione peroxidase shares the substrate with CAT, and it is also designated to be the main protector against oxidative stress at low concentrations of free radicals [23]. Regardless of the importance of analyzing the presence of individual salivary antioxidants, the determination of the total antioxidant capacity of saliva (TAC), as the degree of its total antioxidant ability, is the more informative parameter of this protective property of saliva [24,25]. However, it should be considered that the salivary antioxidant system cannot be seen as a simple sum of the activities of different antioxidant substances, but as a dynamic, complex system of interdependent individual antioxidant enzymes [26].

With the growing interest in the role of oxidative stress in the etiopathogenesis of oral diseases, especially periodontal disease, importance is given to its possible predictive value in the diagnosis of periodontal disease risk [12,27,28]. However, there is very little knowledge on this topic in the child and young adolescent population. Therefore, the primary aim of this study was to examine the antioxidant activity of saliva by determining the TAC value and activity of antioxidant enzymes—CAT and GPX in children and young teenagers without and with gingivitis. The secondary aim was to determine the correlation between these parameters in these two groups.

## 2. Materials and Methods

The research participants were children and young teenagers from an elementary school in Niš, Serbia. All of them were patients of the school’s dental practice, organizational unit of the Service for Preventive and Pediatric Dentistry, Clinic for Dental Medicine, Faculty of Medicine, University of Niš. Written consent for conducting the research was obtained from the school authorities and parents, with the consent of the children and young teenagers, after being informed about the goals of the research. The research was approved by the Ethics Committee, Faculty of Medicine, University of Niš (No: 01-1829, 26 March 2008), and it was in compliance with the principles of the Helsinki Declaration.

The basic conditions for including subjects in the research were: recently completed permanent dentition, absence of active carious lesions (in case of presence, dental fillings had to be older than a month), and absence of acute and chronic general diseases. Whether the child meets these criteria was verified in the dental records and from the questionnaires on the child’s health condition, completed by the parents.

The examination of the gingival condition and saliva sampling were performed in the school’s dental office. The analysis of samples was performed at the Institute of Biochemistry of the Faculty of Medicine, University of Niš, Serbia.

The Löe and Silness Gingival Index was used to assess the condition of the gingiva [29]. The assessment was performed by two researchers, a specialist and a doctor specializing in Preventive and Pediatric Dentistry. Although both examiners were experienced in this kind of examination, they were additionally provided with detailed oral instructions before the beginning of the study. In the case of a dilemma, consensus decision was made.

According to the mentioned index, the clinical examination of the gingiva included the assessment of the condition of the gingiva by inspection, palpation and probing on all four sides of each tooth present. The color, size, consistency, and existence of spontaneous bleeding of the gingiva and swelling were determined by inspection and palpation. The presence or absence of gingival bleeding on provocation were determined with a probe with a rounded tip (Goldman-Fox, Hu-Friedy, Mfg Co., Inc., Chicago, IL, USA) that was placed parallel to the longitudinal axis of the tooth, and the force used for probing was equal to the weight of the probe.

The numerical expression of the gingival condition was performed by scoring a healthy, pale-pink, firm and fine-grained gingival surface with 0. Mildly inflamed gingiva was scored with 1, which signified a slight change in color, and little change in texture. Moderately inflamed gingiva was scored with 2, which signified moderate glazing, redness, oedema, gingival enlargement and bleeding on pressure. Highly inflamed gingiva with prominent redness and gingival enlargement, as well as a tendency towards spontaneous bleeding was scored with 3 [29]. To obtain the total gingival index for each participant, the obtained sum of the scores of the gingival condition from all four tooth sides of all teeth was divided by 4, and this value was further divided by the number of teeth present in the oral cavity.

Participants with gingival inflammation (gingival index 0.1 to 3.0) formed a study group based on the total gingival index for each person. Participants without signs of gingival inflammation (gingival index 0) formed a control group.

Unstimulated saliva was collected for 5 to 10 min in sterile laboratory bottles. All the saliva samples were taken between 8 and 9 a.m., before breakfast and brushing teeth, in order to avoid qualitative and quantitative differences in the saliva composition. The samples were stored on 2 °C and transported within 1 h to the Institute of Biochemistry of the Faculty of Medicine, University of Niš, for further analysis. They were centrifugated on 10,000 rpm on 4 °C for 10 min. Supernatants were separated and stored on −82 °C.

The method according to Koracevic et al. was used for determining the TAC of saliva [30]. It is based on the reaction between the standard solution of the Fe-EDTA complex and hydrogen peroxide, forming an OH radical in the Fenton reaction. The OH radical further degrades benzoate to form TBARS. Antioxidants present in human fluids, including saliva, suppress the production of TBARS, resulting in a color reduction that is measured spectrophotometrically, and defined as antioxidant activity, expressed in μmol/L.

CAT activity was measured using the previously described spectrophotometric method [31], based on the ability of hydrogen peroxide to form stable, colored complexes with molybdenum salts. The reaction was started by the addition of ammonium molybdate, and the intensity of the resulting yellow color was measured according to the control sample [31].

GPX activity was determined spectrophotometrically using the previously described methodology [32]. The principle of the determination of GPX activity was based on the oxidation of glutathione in the presence of tetra butyl hydroperoxide. The color reaction occurs in the reaction of the SH group and DTNB, with the formation of thionitrophenol anion [32].

Statistical data processing was conducted using software package SPSS 14.0. The examined parameters were continuous variables represented by mean values and standard deviations. The Chi-squared test was used for categorical data. Testing the normality of the distribution of continuous parameters was performed using the Shapiro–Wilk test. The comparison of the values of the parameters between the two groups for normal distribution was performed with Student *t* test, and for distributions that deviate from normal ones, the Mann–Whitney test was performed.

Spearman’s correlation rank test was used to determine the correlation of the examined parameters. Values of *p* < 0.05 indicate a statistically significant difference.

Post hoc power analysis was performed. The estimated power study for the group with and without gingival inflammation was above 95%, based on the values of TAC. The power study analysis was performed using G∗power version 3.1.9.2 (Franz Faul, Universitat Kiel, Germany).

## 3. Results

### 3.1. Sample Characteristics

A total of 120 subjects, with a mean age of 12.2 and equal gender representation, were included in the research. Subjects with and without gingivitis did not differ in gender structure or in terms of mean age (Table 1).

The control group consisted of subjects without gingivitis, with a gingival index of 0. In the group of subjects with gingivitis, the mean value of the gingival index was 1.42 ± 0.97 (Table 2).

### 3.2. Descriptive Analysis of TAC of Saliva and Antioxidant Enzymes—CAT and GPX

Comparing the values of TAC and the antioxidant enzymes of saliva—CAT and GPX in subjects with and without gingivitis— the Mann–Whitney test showed statistically significantly higher values of TAC (*p* < 0.001) and CAT activity (*p* < 0.001) in the group of subjects without gingivitis, when compared to the study group with gingivitis (Table 3).

### 3.3. Correlation Analysis

**Figure 1:**
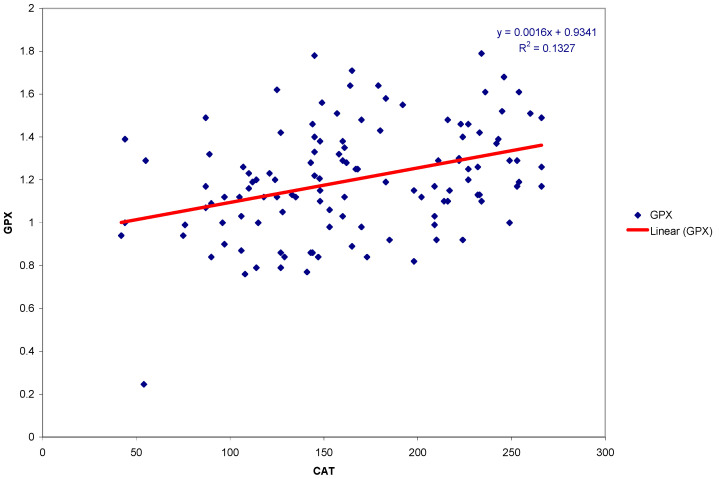
Scatter plot and regression line of GPX and CAT activities in the group of subjects with gingivitis.

The results of determining the interrelationships between TAC of the saliva and antioxidant enzymes CAT and GPX are shown in Table 4 and Table 5 and Figure 1.

The correlation analysis of TAC and antioxidant enzymes in the entire population of subjects showed that there is a statistically significant positive correlation between all parameters, as demonstrated in Table 4.

The correlation analysis of TAC and antioxidant enzymes of saliva in groups of subjects with and without gingivitis is shown in Table 5. In the group of subjects without gingivitis, there is a statistically significant positive correlation only between CAT and GPX activities. In the group of subjects with gingivitis, there is a significant positive correlation between all antioxidant parameters.

The scatter plot and linear regression of GPX and CAT activity in the group of subjects with gingivitis are presented in Figure 1.

## 4. Discussion

This study showed lower values of salivary TAC and the activity of antioxidant enzyme CAT in children and young teenagers with gingivitis, while the GPX was not significantly different. Additionally, there was a strong correlation between all analyzed parameters in group of subjects with gingivitis.

Numerous studies on adult patients have proven the relationship between periodontitis and gingivitis and compromised local TAC [26]. However, studies investigating TAC in relation to periodontal disease in children are scarce [33,34]. They mainly refer to children with existing general disease, most often diabetes and leukemia, which can significantly affect the health of the periodontium and other oral tissues, just as the medications used in the treatment of these diseases, resulting in changes in the saliva composition and TAC values [35,36,37,38]. In addition to there being a small number of studies, their findings are often contradictory, which can be explained by different ways of saliva sampling and storage, different methodologies used in the studies, as well as the method of periodontal disease categorization [26]. With the increasing age of patients and progression of the disease, diagnosis and disease categorization are much easier [39].

In this study, the relationship between salivary TAC and gingival inflammation in children was examined using the Löe–Silness gingival index [29]. Previously, Tóthová et al. examined the TAC value of unstimulated saliva in children using the oral hygiene index (OHI) and papillary bleeding index (PBI), observing a significant correlation between these parameters and TAC values, similar to our study [34]. Aral et al. did not show the change of TAC of the unstimulated saliva in children with gingivitis [33]. This group of authors obtained similar results by analyzing the TAC values in gingival fluid and serum of children with and without gingivitis [33].

Most studies on adults showed reduced TAC values in subjects with periodontal disease, when compared to healthy control groups, in both stimulated [24,26,40,41,42] and unstimulated saliva samples [26,43]. It was also proven that the TAC value of the stimulated saliva is 40 to 50% higher in healthy subjects, compared with subjects with a periodontal disease [43]. However, there are studies with contradictory results, showing no statistically significant decreases in TAC values in subjects with periodontitis, in stimulated [44,45,46] as well as in unstimulated saliva samples [45], or even reporting an increase in salivary TAC values in patients with periodontitis [47].

Current research in the field of risk assessment and monitoring of periodontal disease recognizes saliva as an important biological material for the early detection of specific markers of oxidative stress [26,48]. Considering the simple and non-invasive sampling method, it is especially suitable for use in children and also in a young adolescent group, characterized by higher susceptibility to gingival inflammation due to high hormonal activity in these age [49]. In this study, unstimulated saliva was used, because an unstimulated saliva flow is considered to be the main and basic intraoral condition, thus providing a more realistic presentation of the salivary antioxidant capacity values than stimulated saliva [43]. The influence of exogenous antioxidants introduced by food on the TAC value is mentioned as a possible disadvantage of unstimulated saliva [37]. This possibility was avoided here by taking saliva samples before breakfast and brushing teeth.

Studies examining the association of antioxidant enzymes CAT and GPX with periodontal disease are scarce [18,41,50,51,52], and to our knowledge, this is the first study examining these parameters in children and young teenagers with gingivitis. The examination of CAT in the saliva of our subjects showed a significantly lower mean value in the group of subjects with gingivitis, which indicates impaired antioxidant protection of the inflamed gingiva. Similar observations are noted in adult patients. Trivedi et al. [50] have shown a significant decrease in the CAT activity in the saliva of adult patients with periodontitis, interpreting reduced values by CAT consumption during oxidative stress. Canakci et al. and Miricescu et al. examined the GPX values in patients with chronic periodontitis, interpreting significantly lower values as an impaired antioxidant protection [41,51]. On the other hand, Tsai et al. did not observe a significant difference in the GPX values in healthy patients and those with periodontitis, similar to our study [52].

The correlation analysis of salivary TAC and antioxidant enzymes—CAT and GPX—showed a positive correlation in the total population of subjects, as well as in groups of subjects without gingival inflammation and with gingival inflammation, indicating a significant share of CAT and GPX in salivary TAC. In the mentioned groups of subjects, a significant positive correlation was found between CAT and GPX, which speaks of the synchronicity of their activity in relation to the same substrate, confirmed in other studies [18]. Activity of enzymatic antioxidants may differ during periodontitis, and that difference may result from the initial mobilization of antioxidant reserves, which leads to an increase in the activity of antioxidant enzymes [18].

## 5. Conclusions

A significant change in these antioxidant parameters in saliva in gingivitis, which can be considered as the earliest phase of periodontal disease, indicates the possibility of their further consideration as important biomarkers for the early detection of oxidative stress, indicating a need for further research in this direction.

## Figures and Tables

**Table 1:** Gender and age structure of study participants with and without gingivitis.

Study Subjects	*N*	Females	Males	Age
Without gingivitis	30	16 (53.33%)	14 (46.77%)	12.32 ± 3.28
With gingivitis	90	47 (52.22%)	43 (47.78%)	11.92 ± 2.92

**Table 2:** The value of the gingival index in subjects with and without gingivitis.

Study Subjects	*N*	Gingival Index
Without gingivitis	30	0
With gingivitis	90	1.42 ± 0.97

Data are given as the mean value ± standard deviation.

**Table 3:** Values of total antioxidant capacity of saliva (TAC) and antioxidant enzymes catalase (CAT) and glutathione peroxidase (GPX) in the saliva of subjects with and without gingivitis.

Parameter	Without Gingivitis	With Gingivitis
TAC (μmol/L)	2.84 ± 0.03 ***	2.71 ± 0.18
CAT (U/L)	214.73 ± 31.62 ***	147.69 ± 54.05
GPX (U/L)	1.20 ± 0.23	1.21 ± 0.25

Data are given as the mean value ± standard deviation. *** *p* < 0.001 (Mann–Whitney test).

**Table 4:** Correlation of salivary total antioxidant capacity of saliva (TAC) and antioxidant enzymes catalase (CAT) and glutathione peroxidase (GPX) in the total study population.

ρ	GPX	TAC
CAT	0.367 **	0.464 **
GPX		0.309 **

ρ—Spearman’s rank correlation coefficient. ** *p* < 0.01.

**Table 5:** Correlation of salivary total antioxidant capacity of saliva (TAC) and antioxidant enzymes— catalase (CAT) and glutathione peroxidase (GPX) in the group of subjects with and without gingivitis (Spearman’s rank correlation coefficient).

	Without Gingivitis		With Gingivitis	
Ρ	GPX	TAC	GPX	TAC
CAT	0.430 *	0.109	0.442 **	0.383 **
GPX		0.297		0.349 **

ρ—Spearman’s rank correlation coefficient. * *p* < 0.05; ** *p* < 0.01.

## Data Availability

Additional data that support the findings of this study are available from the corresponding author O.T.J., upon reasonable request.

## References

[1] Mittal M., Siddiqui M.R., Tran K., Reddy S.P., Malik A.B. (2014). Reactive oxygen species in inflammation and tissue injury. Antioxid. Redox Signal..

[2] Vassalle C., Maltinti M., Sabatino L. (2020). Targeting Oxidative Stress for Disease Prevention and Therapy: Where Do We Stand, and Where Do We Go from Here. Molecules.

[3] Nandi A., Yan L.J., Jana C.K., Das N. (2019). Role of Catalase in Oxidative Stress- and Age-Associated Degenerative Diseases. Oxid. Med. Cell Longev..

[4] Zuo L., Wijegunawardana D. (2021). Redox Role of ROS and Inflammation in Pulmonary Diseases. Adv. Exp. Med. Biol..

[5] Janciauskiene S. (2020). The Beneficial Effects of Antioxidants in Health and Diseases. Chronic Obs. Pulm. Dis..

[6] Puglia C.D., Powell S.R. (1984). Inhibition of cellular antioxidants: A possible mechanism of toxic cell injury. Environ. Health Perspect..

[7] Wei Y., Zhang J., Xu S., Peng X., Yan X., Li X., Wang H., Chang H., Gao Y. (2018). Controllable oxidative stress and tissue specificity in major tissues during the torpor-arousal cycle in hibernating Daurian ground squirrels. Open Biol..

[8] Greabu M., Battino M., Mohora M., Totan A., Didilescu A., Spinu T., Totan C., Miricescu D., Radulescu R. (2009). Saliva--a diagnostic window to the body, both in health and in disease. J. Med. Life.

[9] Maciejczyk M., Zalewska A., Ładny J.R. (2019). Salivary Antioxidant Barrier, Redox Status, and Oxidative Damage to Proteins and Lipids in Healthy Children, Adults, and the Elderly. Oxid. Med. Cell Longev..

[10] Greabu M., Battino M., Mohora M., Totan A., Spinu T., Totan C., Didilescu A., Duţa C. (2007). Could constitute saliva the first line of defence against oxidative stress?. Rom. J. Intern. Med..

[11] Sardaro N., Della Vella F., Incalza M.A., DI Stasio D., Lucchese A., Contaldo M., Laudadio C., Petruzzi M. (2019). Oxidative Stress and Oral Mucosal Diseases: An Overview. In Vivo.

[12] Khodaii Z., Mehrabani M., Rafieian N., Najafi-Parizi G.A., Mirzaei A., Akbarzadeh R. (2019). Altered levels of salivary biochemical markers in periodontitis. Am. J. Dent..

[13] Tricković-Janjić O., Cvetković T., Apostolović M., Kojović D., Kostadinović L., Igić M., Surdilović D. (2009). Analysis of enzyme activity and the level of malondialdehyde in the saliva of children with gingivitis. Vojn. Pregl..

[14] Kamodyová N., Tóthová L., Celec P. (2013). Salivary markers of oxidative stress and antioxidant status: Influence of external factors. Dis. Markers.

[15] Nagler R.M., Klein I., Zarzhevsky N., Drigues N., Reznick A.Z. (2002). Characterization of the differentiated antioxidant profile of human saliva. Free Radic. Biol. Med..

[16] Battino M., Ferreiro M.S., Gallardo I., Newman H.N., Bullon P. (2002). The antioxidant capacity of saliva. J. Clin. Periodontol..

[17] Omidpanah N., Ebrahimi S., Raygani A.V., Mozafari H., Rezaei M. (2020). Total Antioxidant Capacity, Catalase Activity and Salivary Oxidative Parameters in Patients with Temporomandibular Disorders. Front. Dent..

[18] Toczewska J., Konopka T. (2019). Activity of enzymatic antioxidants in periodontitis: A systematic overview of the literature. Dent. Med. Probl..

[19] Karincaoglu Y., Batcioglu K., Erdem T., Esrefoglu M., Genc M. (2005). The levels of plasma and salivary antioxidants in the patient with recurrent aphthous stomatitis. J. Oral Pathol. Med..

[20] Damirchi A., Saati Zareei A., Sariri R. (2015). Salivary antioxidants of male athletes after aerobic exercise and garlic supplementation on: A randomized, double blind, placebo-controlled study. J. Oral Biol. Craniofac. Res..

[21] Dabrowski A., Gabryelewicz A. (1992). Oxidative stress. An early phenomenon characteristic of acute experimental pancreatitis. Int. J. Pancreatol..

[22] Narayanankutty A., Job J.T., Narayanankutty V. (2019). Glutathione, an Antioxidant Tripeptide: Dual Roles in Carcinogenesis and Chemoprevention. Curr. Protein Pept. Sci..

[23] Imai H., Nakagawa Y. (2003). Biological significance of phospholipid hydroperoxide glutathione peroxidase (PHGPx, GPx4) in mammalian cells. Free Radic. Biol. Med..

[24] Chapple I.L., Mason G.I., Garner I., Matthews J.B., Thorpe G.H., Maxwell S.R., Whitehead T.P. (1997). Enhanced chemiluminescent assay for measuring the total antioxidant capacity of serum, saliva and crevicular fluid. Ann. Clin. Biochem..

[25] Wang Y., Andrukhov O., Rausch-Fan X. (2017). Oxidative Stress and Antioxidant System in Periodontitis. Front. Physiol..

[26] Toczewska J., Maciejczyk M., Konopka T., Zalewska A. (2020). Total Oxidant and Antioxidant Capacity of Gingival Crevicular Fluid and Saliva in Patients with Periodontitis: Review and Clinical Study. Antioxidants.

[27] Wang J., Schipper H.M., Velly A.M., Mohit S., Gornitsky M. (2015). Salivary biomarkers of oxidative stress: A critical review. Free Radic. Biol. Med..

[28] Podzimek S., Vondrackova L., Duskova J., Janatova T., Broukal Z. (2016). Salivary Markers for Periodontal and General Diseases. Dis. Markers.

[29] Loe H., Silness J. (1963). Periodontal disease in pregnancy. I. Prevalence and severity. Acta Odontol. Scand..

[30] Koracevic D., Koracevic G., Djordjevic V., Andrejevic S., Cosic V. (2001). Method for the measurement of antioxidant activity in human fluids. J. Clin. Pathol..

[31] Koroliuk M.A., Ivanova L., Maĭorova I., Tokarev V. (1988). A method of determining catalase activity. Lab. Delo..

[32] Moin V.M. (1986). A simple and specific method for determining glutathione peroxidase activity in erythrocytes. Lab. Delo..

[33] Aral C.A., Nalbantoğlu Ö., Nur B.G., Altunsoy M., Aral K. (2017). Metabolic control and periodontal treatment decreases elevated oxidative stress in the early phases of type 1 diabetes onset. Arch. Oral Biol..

[34] Tóthová L., Celecová V., Celec P. (2013). Salivary markers of oxidative stress and their relation to periodontal and dental status in children. Dis. Markers.

[35] Baliga S., Chaudhary M., Bhat S., Bhansali P., Agrawal A., Gundawar S. (2018). Estimation of malondialdehyde levels in serum and saliva of children affected with sickle cell anemia. J. Indian Soc. Pedod. Prev. Dent..

[36] Fathi S., Borzouei S., Goodarzi M.T., Poorolajal J., Ahmadi-Motamayel F. (2020). Evaluation of Salivary Antioxidants and Oxidative Stress Markers in Type 2 Diabetes Mellitus: A Retrospective Cohort Study. Endocr. Metab. Immune Disord. Drug Targets.

[37] Żukowski P., Maciejczyk M., Waszkiel D. (2018). Sources of free radicals and oxidative stress in the oral cavity. Arch. Oral Biol..

[38] Hegde A.M., Joshi S., Rai K., Shetty S. (2011). Evaluation of oral hygiene status, salivary characteristics and dental caries experience in acute lymphoblastic leukemic (ALL) children. J. Clin. Pediatr. Dent..

[39] Tonetti M.S., Greenwell H., Kornman K.S. (2018). Staging and grading of periodontitis: Framework and proposal of a new classification and case definition. J. Periodontol..

[40] Baltacıoğlu E., Kehribar M.A., Yuva P., Alver A., Atagün O.S., Karabulut E., Akalın F.A. (2014). Total oxidant status and bone resorption biomarkers in serum and gingival crevicular fluid of patients with periodontitis. J. Periodontol..

[41] Miricescu D., Totan A., Calenic B., Mocanu B., Didilescu A., Mohora M., Spinu T., Greabu M. (2014). Salivary biomarkers: Relationship between oxidative stress and alveolar bone loss in chronic periodontitis. Acta Odontol. Scand..

[42] Tripathi V., Singh S.T., Sharma V., Verma A., Singh C.D., Gill J.S. (2018). Assessment of Lipid Peroxidation Levels and Total Antioxidant Status in Chronic and Aggressive Periodontitis Patients: An in vivo Study. J. Contemp. Dent. Pract..

[43] Diab-Ladki R., Pellat B., Chahine R. (2003). Decrease in the total antioxidant activity of saliva in patients with periodontal diseases. Clin. Oral Investig..

[44] Ahmadi-Motamayel F., Goodarzi M.T., Jamshidi Z., Kebriaei R. (2017). Evaluation of Salivary and Serum Antioxidant and Oxidative Stress Statuses in Patients with Chronic Periodontitis: A Case-Control Study. Front. Physiol..

[45] Brock G.R., Butterworth C.J., Matthews J.B., Chapple I.L. (2004). Local and systemic total antioxidant capacity in periodontitis and health. J. Clin. Periodontol..

[46] Novakovic N., Todorovic T., Rakic M., Milinkovic I., Dozic I., Jankovic S., Aleksic Z., Cakic S. (2014). Salivary antioxidants as periodontal biomarkers in evaluation of tissue status and treatment outcome. J. Periodontal Res..

[47] Almerich-Silla J.M., Montiel-Company J.M., Pastor S., Serrano F., Puig-Silla M., Dasí F. (2015). Oxidative Stress Parameters in Saliva and Its Association with Periodontal Disease and Types of Bacteria. Dis. Markers.

[48] Antezack A., Chaudet H., Tissot-Dupont H., Brouqui P., Monnet-Corti V. (2020). Rapid diagnosis of periodontitis, a feasibility study using MALDI-TOF mass spectrometry. PLoS ONE.

[49] Hosadurga R., Nabeel Althaf M.S., Hegde S., Rajesh K.S., Arun Kumar M.S. (2016). Influence of sex hormone levels on gingival enlargement in adolescent patients undergoing fixed orthodontic therapy: A pilot study. Contemp. Clin. Dent..

[50] Trivedi S., Lal N., Mahdi A.A., Singh B., Pandey S. (2015). Association of salivary lipid peroxidation levels, antioxidant enzymes, and chronic periodontitis. Int. J. Periodontics Restor. Dent..

[51] Canakci C.F., Cicek Y., Yildirim A., Sezer U., Canakci V. (2009). Increased levels of 8-hydroxydeoxyguanosine and malondialdehyde and its relationship with antioxidant enzymes in saliva of periodontitis patients. Eur. J. Dent..

[52] Tsai C.C., Chen H.S., Chen S.L., Ho Y.P., Ho K.Y., Wu Y.M., Hung C.C. (2005). Lipid peroxidation: A possible role in the induction and progression of chronic periodontitis. J. Periodontal Res..

